# Joint denoising and motion correction: initial application in single-shot cardiac MRI

**DOI:** 10.1186/1532-429X-17-S1-Q29

**Published:** 2015-02-03

**Authors:** Aurelien Bustin, Martin A Janich, Anja C Brau, Freddy Odille, Steven D Wolff, Oleg Shubayev, David Stanley, Anne Menini

**Affiliations:** 1 GE Global Research, Garching, Germany; 2 Computer Science, Technische Universität München, München, Germany; 3 Cardiac Center of Excellence, GE Healthcare, Garching, Germany; 4 U947, INSERM, Nancy, France; 5 Advanced Cardiovascular Imaging, Carnegie Hill Radiology, New York City, NY, USA; 6 Imagerie Adaptative Diagnostique et Interventionnelle, University de Lorraine, Nancy, France; 7 GE Healthcare, Rochester, MN, USA

## Background

Single-shot (SSH) pulse sequences in CMR are beneficial for rapid image acquisition that is robust to motion, especially in arrhythmic patients or poor breath-holders. However, this fast scanning technique trades scan time for a lower signal-to-noise ratio compared to conventional multi-shot acquisitions. Here we propose a motion-compensated denoising technique that improves the image quality from multiple free-breathing single-shot acquisitions.

## Methods

Acquisition - In 7 patients with suspected cardiovascular disease, 4 repetitions of SSH Late Gadolinium Enhancement (LGE) were acquired in free-breathing 7-10 minutes after Gd injection [1]. The experiments were performed on 3T MR750w and 1.5T MR450w systems (GE Healthcare, WI, USA) with the following parameters: 8-mm slice, acceleration factor=2. The scan time of SSH LGE imaging (8s/slice in free-breathing) was shorter than the multi-shot LGE (13s/slice in breath-hold).

Reconstruction - The proposed method consists of 3 steps: 1) SENSE reconstruction of the undersampled data, 2) extraction of the motion that occurred between the repetitions with a non-rigid registration, and 3) motion compensated joint denoising of the whole dataset with Beltrami regularization, which offers an ideal compromise between feature preservation and staircasing reduction [2]. Steps 2 and 3 are applied iteratively. The results were compared to the non-iterative previously proposed method [3] where the third step is replaced by a simple average of the registered images.

## Results

**Figure 1:**
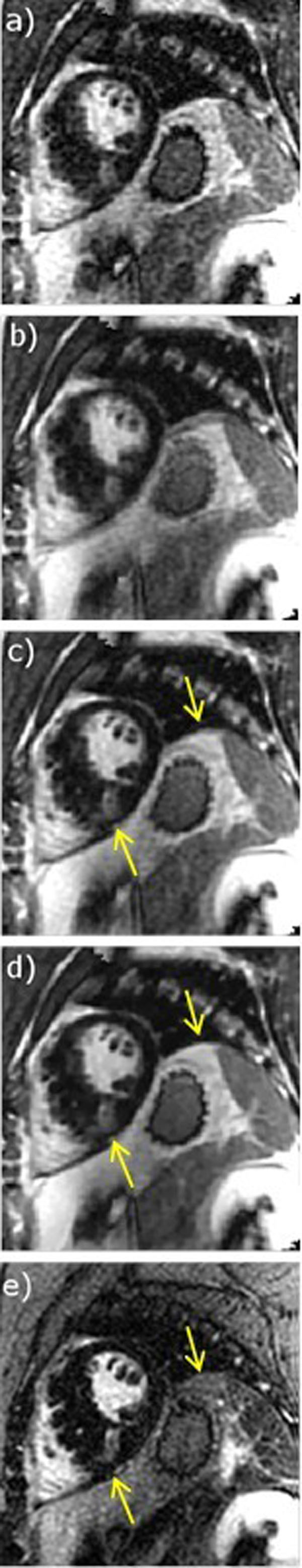
Cardiac imaging reconstructions after SSH LGE acquisition in free breathing. a) One Repetition (192x152), b) Average of 4 Repetitions, c) Iterative motion-compensated reconstruction with averaging instead of denoising, d) Iterative Reconstruction with Beltrami regularization. e) Conventional multi-shot LGE in breath-hold (224x192).

Example reconstructions results are shown in Fig 1. Images reconstructed by employing a standard averaging of 4 SSH LGE images acquired during free-breathing show a result spatially blurred and noisy (b). The benefit of motion correction is seen on (c), and the benefit of denoising in (d). Compared to the non-iterative method, one can see that the proposed joint denoising and motion correction method reconstructs sharper edges (arrows) while reducing the noise.

## Conclusions

We introduced a new joint denoising and motion correction algorithm designed for fast, robust cardiac imaging. By iteratively incorporating the estimated motion into the reconstruction process, we increase the robustness of the model and exhibit good quality imaging. A limitation to the method is that potential through-plane motion cannot be corrected, although it remains negligible compared to the slice thickness. Ultimately, this method could be beneficial to both cardiac exam workflow and patient comfort since it allows robust image quality without the need for breath-holds.

## Funding

This work was supported by the European Commission, through Grant 605162.
